# Transcript profiling reveals complex auxin signalling pathway and transcription regulation involved in dedifferentiation and redifferentiation during somatic embryogenesis in cotton

**DOI:** 10.1186/1471-2229-12-110

**Published:** 2012-07-20

**Authors:** Xiyan Yang, Xianlong Zhang, Daojun Yuan, Fangyan Jin, Yunchao Zhang, Jiao Xu

**Affiliations:** 1 National Key Laboratory of Crop Genetic Improvement, Huazhong Agricultural University, Wuhan, Hubei, 430070, P. R. China

**Keywords:** Auxin signalling pathway, Cotton, RNA-seq, Somatic embryogenesis, Transcription profile, Transcription regulation

## Abstract

**Background:**

Somatic embryogenesis (SE), by which somatic cells of higher plants can dedifferentiate and reorganize into new plants, is a notable illustration of cell totipotency. However, the precise molecular mechanisms regulating SE remain unclear. To characterize the molecular events of this unique process, transcriptome analysis, in combination with biochemical and histological approaches, were conducted in cotton, a typical plant species in SE. Genome-wide profiling of gene expression allowed the identification of novel molecular markers characteristic of this developmental process.

**Results:**

RNA-Seq was used to identify 5,076 differentially expressed genes during cotton SE. Expression profile and functional assignments of these genes indicated significant transcriptional complexity during this process, associated with morphological, histological changes and endogenous indole-3-acetic acid (IAA) alteration. Bioinformatics analysis showed that the genes were enriched for basic processes such as metabolic pathways and biosynthesis of secondary metabolites. Unigenes were abundant for the functions of protein binding and hydrolase activity. Transcription factor–encoding genes were found to be differentially regulated during SE. The complex pathways of auxin abundance, transport and response with differentially regulated genes revealed that the auxin-related transcripts belonged to IAA biosynthesis, indole-3-butyric acid (IBA) metabolism, IAA conjugate metabolism, auxin transport, auxin-responsive protein/indoleacetic acid-induced protein (Aux/IAA), auxin response factor (ARF), small auxin-up RNA (SAUR), Aux/IAA degradation, and other auxin-related proteins, which allow an intricate system of auxin utilization to achieve multiple purposes in SE. Quantitative real-time PCR (qRT-PCR) was performed on selected genes with different expression patterns and functional assignments were made to demonstrate the utility of RNA-Seq for gene expression profiles during cotton SE.

**Conclusion:**

We report here the first comprehensive analysis of transcriptome dynamics that may serve as a gene expression profile blueprint in cotton SE. Our main goal was to adapt the RNA-Seq technology to this notable development process and to analyse the gene expression profile. Complex auxin signalling pathway and transcription regulation were highlighted. Together with biochemical and histological approaches, this study provides comprehensive gene expression data sets for cotton SE that serve as an important platform resource for further functional studies in plant embryogenesis.

## Background

Somatic embryogenesis (SE) is the developmental process by which somatic cells undergo dedifferentiation to generate embryogenic cells and form a somatic embryo, from which new plants can be regenerated [1,2]. This process can be divided into two stages. First, somatic cells re-enter the cell cycle and transform into a dedifferentiated cell state; they then acquire embryogenic potential, characterized by a reorganization of cell physiology, metabolism and gene expression [3]. This process is experimentally induced by changes of culture conditions, using exogenous plant growth regulators (PGRs) and stress. Following that, cells with embryogenic potential can differentiate into somatic embryos [4,5].

Morphological, biochemical, and more recently, molecular aspects of SE have been described [6-8]. Following early preliminary experiments on differential gene expression [9], the mechanisms of gene regulation during SE in many plant species, such as carrot [10], cotton [11], Arabidopsis [12], alfalfa [13], soybean [7] and potato [14] have been investigated. These experiments have resulted in the isolation of numerous genes which are specifically activated, or exhibit differential expression, during SE [1,15].

Cotton is one of the most important economic crops and the main source of natural fibre, but further trait improvement requires efficient genetic manipulation [11]. Cell biological approaches, including tissue culture and genetic engineering, have been widely applied to cotton breeding. As a result, transgenic varieties with herbicide and pest resistance have been developed [16]. However, a reproducible and highly efficient plant regeneration scheme is required for cotton species, which remains a recalcitrant species to manipulate *in vitro*[17]. Thus far, reports of high-frequency regeneration of cotton via SE have been limited owing to a genotype-dependent response [18], and the majority of the reports on *in vitro* regeneration of cotton only pertain to specific varieties such as Coker lines [19]. More recently, an elite genotype for *in vitro* cellular manipulation, which showed more efficient regeneration ability than Coker lines, was identified in our laboratory [20]. The identification and isolation of genes critical for SE are of great importance for improving the embryogenic competence and regenerability of a wider range of cultivars and thus accelerating the production of transgenic cotton varieties. This requires new molecular information.

Physiological and biochemical changes during SE are reflected in the transcriptional modulation of many genes [7,9,21]. A number of genes that are activated or differentially expressed during the induction and development of somatic embryos have been cloned and studied using various molecular techniques [22-24]. However, little is known about global transcriptional changes and their regulation. Genome-wide expression analyses provide essential building blocks for elucidating molecular function. The analysis of cDNA amplified fragment length polymorphisms [25], Suppression Subtractive Hybridization (SSH) and microarrays [11,21,26] have provided the first pictures of transcriptome dynamics during cotton SE and early events of cellular dedifferentiation, but these approaches suffer from a number of drawbacks and the data are far from complete. Recent studies have highlighted the significance of next-generation sequencing technologies for genome-scale expression analyses in higher eukaryotes, including whole-transcript sequencing and assembly (RNA-Seq) using the long-read, 454 platform [27] and the massively parallel Illumina [28] and ABI SOLiD [29] systems. These approaches produce millions of short cDNA reads that can be mapped to a reference genome and/or transcriptome sequence to obtain a genome-scale transcriptional map consisting of the transcriptional structure and the expression level for each gene. Resolution of these networks is possible because of the increased sensitivity and specificity of transcript analysis by the method [30].

To create a more complete survey of transcriptome content and dynamics during cotton SE, we used a next-generation sequencing approach, Illumina Digital Gene Expression (DGE) technology. To our knowledge, this represents the first genome-wide gene expression profiling of SE in cotton, and the data presented here will serve as a foundational resource for future studies addressing fundamental molecular and developmental mechanisms that govern plant embryogenesis.

## Results

### Kinetics of cotton SE

**Figure 1:**
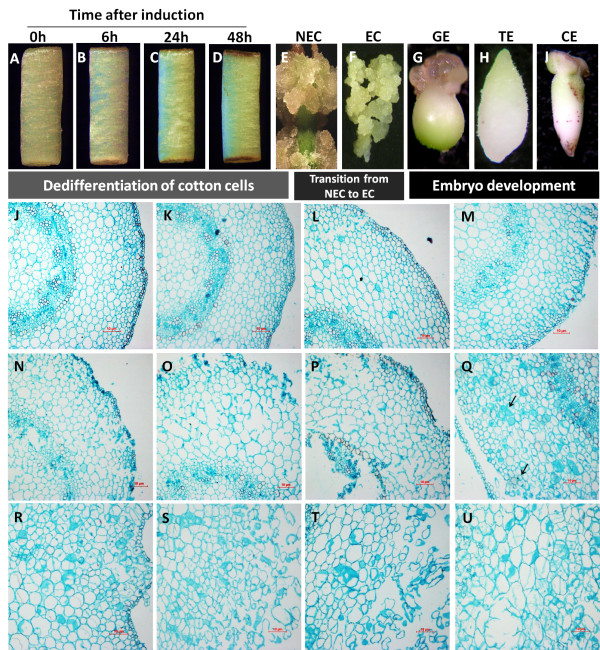
**Schematic representation and histological observation of different time points/stages during somatic embryogenesis used for RNA-Seq analysis.** Initial hypocotyl explants (**A**) used as control. Initial cellular dedifferentiation was sampled from explants after induction for 6 h, 24 h, and 48 h (**B**-**D**). Typical nonembryogenic calli (NECs) were sampled at 40 d of culture time (**E**) when the calluses were loosening and abundant, embryogenic calluses (ECs) were sampled after the first subculture when compact primary embryogenic clumps (**F**) were first identified. Different stages of somatic embryos [globular embryos (GEs), (**G**), torpedo embryos (TEs), (**H**), and cotyledon embryos (CEs), (**I**)] were sampled after synchronization control of somatic embryogenesis by suspension culture. Somatic embryogenesis in cotton passes through three different processes: dedifferentiation of cotton somatic cells, transition from NECs to ECs, and development of Somatic embryos. Histological analysis was made at 0 h, 3 h, 6 h, 12 h, 24 h, 48 h, 72 h, 7 d, 10 d, 15 d, 25 d, and 40 d after induction (**J**-**U**).

Changes in morphology and histology during SE were determined in explants over 40 days following phytohormone induction [indole-3-butyric acid (IBA) + kinetin (KT)]. Hypocotyl explants were used as controls (Figure 1A). To monitor initial cellular dedifferentiation events, explants were sampled at 6 h, 24 h and 48 h after induction (Figure 1B-D). Nonembryogenic calli (NECs) were sampled at 40 d post-induction (Figure 1E) when the calli were loosening, and abundant. Embryogenic calli (ECs) were sampled after one subculture, when compact primary embryogenic clumps were first identified (Figure 1F). Different stages of somatic embryos [globular embryos (GEs) (Figure 1G); torpedo embryos (TEs) (Figure 1H); cotyledon embryos (CEs) (Figure 1I)] were obtained by experimental synchronization of the suspension culture.

Hypocotyls cultured for 3 h showed no morphological changes compared to fresh hypocotyls (0 h explants, Figure 1J-K). On the third day of induction, both ends of hypocotyls had expanded, but histological observation showed expanding epidermal cells at 24 h after induction (Figure 1N). Some epidermal cell expansion was evident by 6 h to 12 h after induction (Figure 1L-M). Subsequently, epidermal, parenchyma and primary cambium cells expanded and dissociated from explants (Figure 1O-P). After 7 d of culture, callus could be seen at the end of explants, which subsequently proliferated. Histological observation showed that the epidermal and primary cambium cells rapidly entered cell division and the unattached meristematic cell masses separated from the primary meristem (Figure 1Q). Some cells went through distinct cellular dedifferentiation and then began to divide and proliferate normally (Figure 1R-U). After about 40 d of culture, some ECs were produced. Thus, SE in cotton passes through three different processes as shown in Figure 1: dedifferentiation of cotton somatic cells, transition from NECs to ECs and development of somatic embryos.

### Dynamics of endogenous indole-3-acetic acid during SE

**Figure 2:**
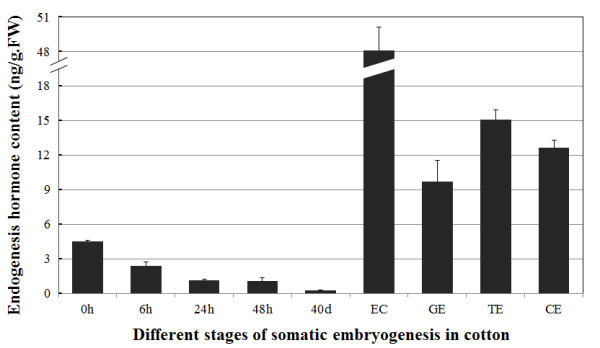
Endogenous IAA content in dedifferentiation and redifferentiation cultures during cotton SE.

To monitor auxin changes during SE, the concentration of endogenous indole-3-acetic acid (IAA) was measured by HPLC-MS at different time points during dedifferentiation and redifferentiation during cotton SE. It was found that the endogenous IAA concentration gradually decreased during dedifferentiation and then increased to reach a relatively high level (11-fold of that in hypocotyl) in ECs (Figure 2). Previous studies have also shown that sharp changes in endogenous auxin levels might be one of the first steps leading to SE [31,32]. However, the endogenous level of IAA subsequently decreased during somatic embryo development and showed modulated concentration in GEs (2.17-fold of that in hypocotyl), TEs (3.37-fold of that in hypocotyl) and CEs (2.82-fold of that in hypocotyl) (Figure 2). Therefore during the dedifferentiation process and subsequent division of callus cells, the IAA content steadily decreased until redifferentiation, while redifferentiation was correlated with a sharp increase in auxin concentration (Figure 2). During the late dedifferentiation (NEC) stage, with the lowest extreme of IAA content (0.04-fold of that in hypocotyl) detected (Figure 2).

### Global analysis of differential gene expression during SE

High resolution analysis of differentially expressed genes during SE was achieved using RNA-Seq technology. A total of 32,108,458 clean tags of 21 bp in length were generated. Sequencing saturation analysis indicated that these were sufficient for quantitative analysis of gene expression ( Additional file 1 Figure S1). There was an average of 1,358,599 unambiguous mapped tags, i.e., 90.25% of all mapped tags (1,505,420), representative of 70,086 distinct unambiguous mapped tags, representing 97.47% of all reference tags (Table 1, Additional file 2Table S1). To reveal the molecular events behind DGE profiles, we mapped the tag sequences to a reference database (cotton unigenes from NCBI) containing 20,671 unigene sequences. Of these sequences, 93.66% (19,360) possessed the *Nla*III restriction site (CATG) used in the tag library construction. For each sample, more than half of the tags could not be mapped to the cotton reference unigenes (Table 1). Among the 116,334 (TEs) to 134,986 (48 h) distinct tags generated from the Illumina sequencing of these libraries, 30,986 (GEs) to 39,970 (48 h) distinct unambiguous tags were mapped to a gene in the reference database ( Additional file 2 Table S1). Up to 50.7% (15,339) of the sequences in our reference database could be unambiguously identified by unique tags ( Additional file 3 Table S2). Tags mapped to a unique sequence are the most critical subset of the DGE libraries because they can unambiguously identify a transcript.

**Table 1:** Overview of tag number

		**No. of clean tag**	**No. (%) of Unambiguous mapped Tag**	**No. (%) of Unambiguous tag-matched unigene**	**No. (%) of unknown tag**
Time of hypocotyls cultured (h)	0	3713673	1551964(41.79)	13446(65.05)	1994178(53.70)
	6	3574921	1361344(38.08)	13513(65.37)	2093782(58.57)
	24	3421294	1393380(40.73)	13781(66.67)	1891802(55.29)
	48	3433665	1239777(36.11)	12595(60.93)	1970515(57.39)
Noembryogenic calli		3649041	1423394(39.01)	12687(61.38)	2013798(55.19)
Embryogenic calli		3542887	1237924(34.94)	12230(59.17)	2185187(61.68)
Globular embryo		3542767	1333449(37.64)	12406(60.02)	2113500(59.66)
Torpedo embryo		3662505	1427168(38.97)	12749(61.68)	2134578(58.28)
Cotyledon embryo		3567705	1258997(35.29)	12649(61.19)	2162331(60.61)
Average		3567606	1358600(38.06)	12895(62.38)	2062186(57.82)

The expression level of genes was determined by calculating the number of unambiguous tags for each gene and then normalizing this to the number of transcripts per million tags (TPM) [33]. An average of 34,278 unambiguous clean tags per sample were calculated for each gene and then normalized to TPM, which linked the tag numbers with gene expression levels. The summary of the tag information and gene expression level is shown in Additional file 3 Table S2. We detected the expression of 15,339 genes during cotton SE. The dynamic range of DGE spanned five orders of magnitude. However, the tag counts for the majority of genes were low in these libraries. Among these, 5,076 differentially expressed genes were filtered with a cut-off of TPM ≥ 20 (*P* ≤ 0.001) and the absolute value of log2Ratio ≥1 based on the false discovery rate (FDR) < 0.05 ( Additional file 4 Table S3).

**Figure 3:**
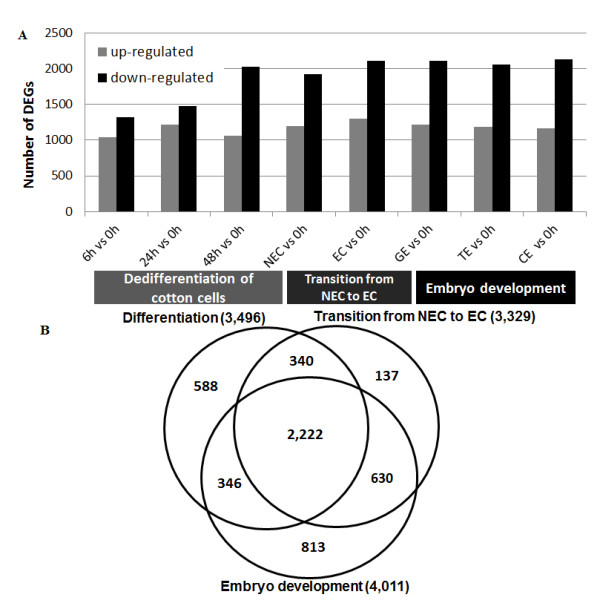
**Histogram and Venn diagram of differentially expressed genes during SE.** The number of genes up- or down-regulated at different time points/stages during SE (**A**) and a Venn diagram showing the differentially expressed genes in each of the three different developmental processes of cotton SE, with the overlapping regions corresponding to the number of differentially expressed genes present in more than one process. The central region corresponds to the expressed genes present in all three processes (**B**).

The number of genes up- or down-regulated at different developmental stages is shown in Figure 3A. Spatial analysis was also performed on differentially expressed genes to ascertain the degree of overlap existing between the three different developmental processes during cotton SE. There were 3,496, 3,329 and 4,011 differentially expressed genes during dedifferentiation, the transition from NECs to ECs and the somatic embryo development process, respectively (Figure 3B). Among these, less than half (43.8%) of the differentially expressed genes were present in all three developmental processes. Significant numbers of genes were present in one developmental process only: 588 genes were only differentially expressed during dedifferentiation of cotton cells, 137 differentially expressed genes were only switched on/off during the transition from NECs to ECs, and 813 genes changed their expression level only during the somatic embryo development process, which suggested that distinct spatial transcriptional profiles were present.

Annotation of these differentially expressed genes (5,076) was first done by searching using BLASTx against the non-redundant protein sequence (nr) database in GenBank using a cut-off E-value of 10^−5^. Using this approach, 3,274 genes (64.50% of all differentially expressed sequences) returned an above cut-off BLAST result ( Additional file 4 Table S3). A further 1,308 genes (25.77%) belonged to the functional category ‘unclassified proteins’ or ‘predicted protein’. 494 differentially expressed genes could not be matched to any genes in the nr protein database. In a reciprocal BLAST search, we also identified 4,536 cotton unigenes (89.36%) that had an ortholog in Arabidopsis ( Additional file 4 Table S3).

To identify the biological pathways that are active during SE in cotton, we mapped the 5,076 annotated sequences to the reference canonical pathways in the Kyoto Encyclopedia of Genes and Genomes (KEGG) [34]. In total, we assigned 2,118 sequences to 256 KEGG pathways ( Additional file 5 Table S4). The pathways with the most representation by unique sequences were ‘metabolic pathways’ (291 members) and ‘biosynthesis of secondary metabolites’ (120 members). A hallmark of dedifferentiation and following somatic embryo development is the ability to control cell division and cell wall accumulation associated with polysaccharides metabolism with corresponding hydrolytic enzymes and their accumulation of storage reserves and secondary metabolites [35]. ‘Spliceosome’ (93 members), ‘microbial metabolism in diverse environments’ (70 members) and ‘oxidative phosphorylation’ (53 members) were also enriched as were 22 genes in the ‘plant hormone signal transduction’ pathway. Given the known role of auxin in regulating plant embryogenesis [4], the competence for embryogenic induction might be the result of regulated auxin responses of these cells. These annotations provide a valuable resource for investigating specific processes, functions and pathways and allow for the identification of novel genes involved in these pathways.

Functional categories were assigned to all predicted genes in terms of gene ontology (GO) [36]. We added GO terms using Blast2GO based on the automated annotation of each unigene using BLAST results against the GenBank nr protein database from NCBI [37]. A total of 3,438 unigenes (67.73%) could be assigned to one or more ontologies, and 2,588 (50.99%) unigenes with assigned GO terms had molecular functions, 2405 (47.38%) were involved in biological processes and 2629 (51.79%) were cellular components; 1657 (32.64%) unique sequences were classified in three ontologies. In each main category, the percentages of different levels do not add up to 100% because some deduced proteins had more than one GO category assigned to them. GO annotations for the differentially expressed genes showed fairly consistent sampling of functional classes.

**Figure 4:**
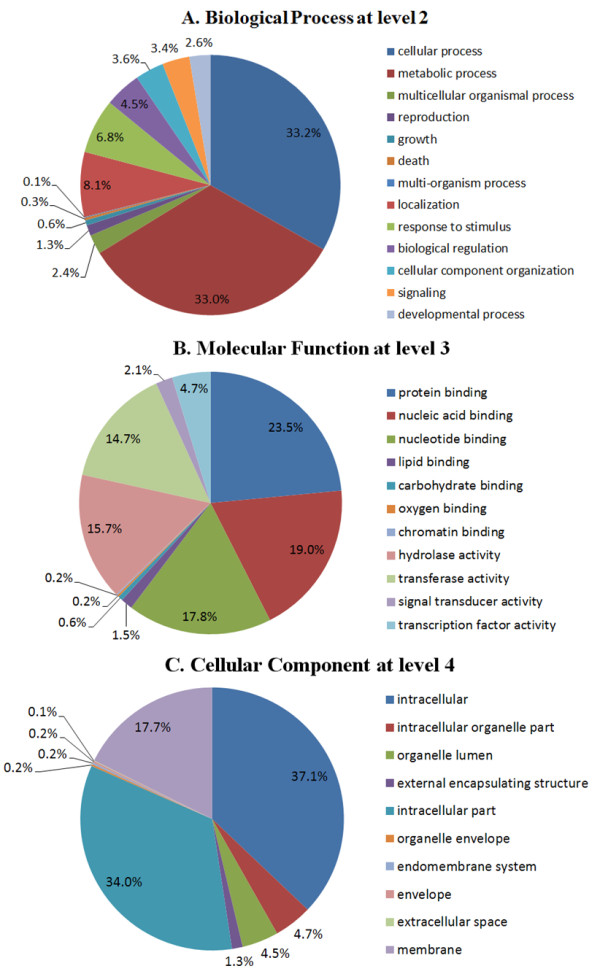
**Functional categories of 5,076 differentially expressed genes that were assigned with GO terms at the appropriate level.** The three GO categories, biological process at level 2 (**A**), molecular function at level 3 (**B**) and cellular component at level 4 (**C**) are presented. The percentages were calculated with respect to all differentially expressed genes in SE

Cellular and metabolic processes were among the most highly represented groups in the biological process category, with each accounting for one third of all genes. This might reflect rapid cell growth and extensive metabolic activities. Genes involved in other important biological processes such as localization (8.1%), response to stimulus (6.8%) and reproduction (4.5%) were also identified (Figure 4A). Under the molecular function category, assignments were mainly to the catalytic and binding activities. In the binding subset, three main groups were present: protein binding (23.5%), nucleic acid binding (19.0%) and nucleotide binding (17.8%). In the catalytic activity subset, two main groups were included: hydrolase activity (14.7%) and transferase activity (15.7%). Transcription factors (4.7%) and signal transducers (2.1%) were also well represented (Figure 4B). The cellular component category identified many genes belonging to the cell part and specifically to intracellular (37.1%) and intracellular organelle parts (34.0%) and the membrane (17.7%) (Figure 4C). A summary of differentially expressed genes annotated in each GO term is shown in Additional file 6 Table S5.

**Figure 5:**
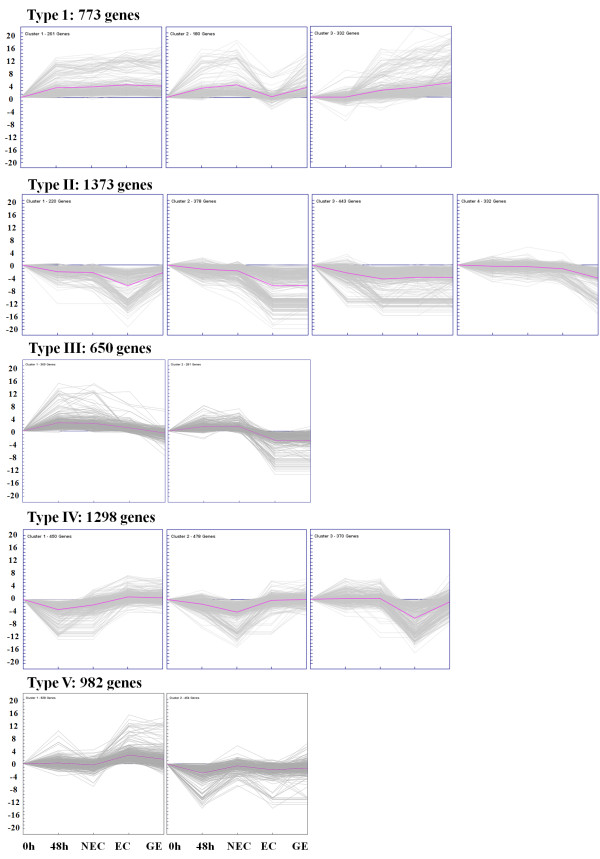
Clustering of differentially expressed genes during somatic embryogenesis in cotton was developed by K-means method based on their expression modulation

During further investigation of the expression profile of these differentially expressed genes, the 5,076 genes were subjected to hierarchical clustering with the k-means method using Pearson’s correlation distance based on their expression modulation. Each gene was assigned to one of five expression types (Figure 5), representing the number of profiles indicated by figure of merit analysis [38]. Type I genes were positively modulated throughout the whole process and were divided into three sub-clusters. Type II genes were negatively modulated throughout the whole process and divided into four sub-clusters based on their expression level in three different processes. Genes in type III up-regulated during dedifferentiation and then down-regulated during EC stage (sub-cluster 1) or somatic embryo development (sub-cluster 2). While, genes in type IV showed a much different manner, they down-regulated and displayed relative low expression level during dedifferentiation (sub-cluster 1) or at NEC stage (sub-cluster 2), and then up-regulated. However, genes in Type V displayed complex expression profiles and fell into two sub-clusters. Sub-cluster 1 contained genes that were up-regulated during the initial dediffer**e**ntiation and showed a relative high peak value at 48 h and then down-regulated at NEC stage, with another high peak value at EC stage, while genes in sub-cluster 2 had expression models opposite that of sub-cluster 1. Expression data for each gene type are available in the Additional file 7 Table S6.

### Transcription Factor mRNA present during cotton SE

**Figure 6:**
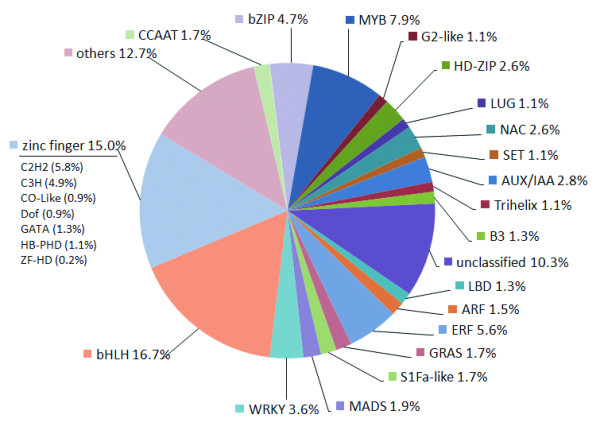
**Differentially expressed TF genes and classification of TF families.** TF genes were classified into TF families by using publicly available Arabidopsis TF databases [Database of Arabidopsis Transcription Factors (DATF); Plant Transcription Factor Database (PlnTFDB); cotton Transcription Factor (http://planttfdb.cbi.pku.edu.cn:9010/web/index.php?sp=gh)]. The 466 TF genes were classified into 22 TF families. Numbers represent the percentage of TF families out of the 466 TF genes. The classification and annotation of all TF genes with respect to functional categories and transcription factor families are presented in Additional file 8 TableS7

From a total of 5,076 differentially expressed genes, 466 TF mRNAs, which were differentially expressed with a wide range in abundance (Figure 6; Additional file 8 Table S7), were identified. The proportion of TF transcripts relative to the total mRNAs within a population was about 9.18% (466 in 5,076). Among those, 127 were up-regulated, and others were down-regulated. More down-regulated TF mRNAs were detected in the somatic embryo development stage compared with the early dedifferentiation stage and the transition from NECs to ECs stage, reflecting a decrease in TF transcription during the late stage of SE. Among these, 16, 15, 11, 10, 20, 48, 31, 26 and 27 TF mRNAs could not be detected at 0 h, 6 h, 24 h, 48 h, NEC, EC, GE, TE, CE time-points/stages, respectively. Collectively, we detected 351 diverse TF mRNAs throughout the dedifferentiation stage (6 h to NEC), 338 during the transition from NECs to ECs and 342 during somatic embryo development stages ( Additional file 8 Table S7).

We annotated features to major TF families to determine the spectrum of TF mRNAs present during this process (Figure 6). All major TF families were represented in the mRNA population at each developmental stage. Taken together, these data suggested that more than 400 diverse TF mRNAs are expressed during SE; the number of TF mRNAs decreased during late embryogenesis; and the representation of specific TF mRNA families differed at specific developmental periods. Zinc finger and bHLH family TFs occupied one third of all TFs. The MYB family TFs, and others such as ERF, bZIP and WRKY, each accounted for 4% to 6% of identified transcripts.

### Analysis of the auxin signalling pathway during cotton SE

Auxin plays a role in dedifferentiation and redifferentiation, through the modulated response, transduction and amplification of the auxin signal to regulate the expression of genes. In this study, 86 genes related to auxin synthesis, transport, metabolism and the signalling pathway were differentially expressed during SE ( Additional file9 Table S8-1). Transcript levels of genes related to auxin homeostasis varied during this process, consistent with a role for auxin in SE. Combined with KEGG results and other annotations, these genes were related to IAA biosynthesis (8 transcripts), IBA metabolism (9 transcripts), IAA conjugate metabolism (8 transcripts), auxin transport (10 transcripts), Aux/IAA (13 transcripts), ARF (6 transcripts), SAUR (4 transcripts), Aux/IAA degradation (17 transcripts) and other auxin-related proteins (11 transcripts).

**Figure 7:**
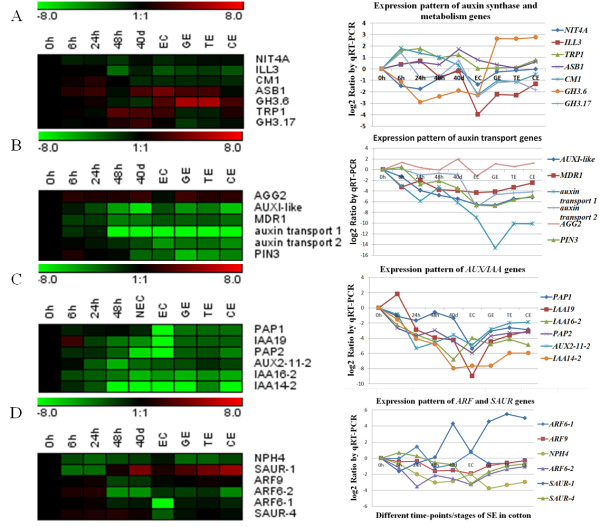
**Detailed expression profiles of genes involved in auxin biosynthesis and signalling pathway.** The relative expression level was obtained by RNA-Seq after taking equation and logarithmic transformations of TPM and by qRT-PCR for data verification

Eight IAA biosynthesis transcripts, encoding tryptophan biosynthesis 1 (*TRP1*), anthranilate synthase (*ASB1*), tryptophan synthase β-subunit 2 (*TSB2*), nitrilase 4 (*NIT4*), chorismatemutase (*CM1*), *CYP79C1*, *YUC* and *FMO*, showed a complex expression pattern throughout the cotton SE process. *TRP1* and *ASB1* were up-regulated throughout embryogenesis, while *NIT4A* was down-regulated, a pattern which was also confirmed by qRT-PCR analysis (Figure 7A). Transcripts for *NIT4A* and *CM1* were restricted to dedifferentiating cells ( Additional file 9 Table S8-2), while transcripts for *CYP79C1* and *YUC* were restricted to embryogenic tissues ( Additional file 9 Table S8-4). Transcripts associated with IAA conjugate metabolism, such as IAA-amino acid conjugate hydrolase/metallopeptidase (*ILL3, ILL6*), IAA amidosynthetase (*GH3.6*, *GH3.17*), IAA-leucine resistant (*ILR1*, *ILR3*) and IAA carboxylmethylransferase 1 (*IAMT1*), were down-regulated. Some transcripts for IBA metabolism were also modulated ( Additional file 9 Table S8-1).

Ten auxin transport-associated transcripts were differentially expressed. Three *AUX1* homologous transcripts (zhu1_Ghi#S33799879, zhu1_Ghi#S42308191 and zhu1_Ghi#S29994266) were down-regulated during cotton SE, while only *AUXI*-*LIKE* displayed high levels ( Additional file 9 Table S8-1). Like *AUX1*, three transcripts (zhu1_Ghi#S33805308, zhu1_Ghi#S42333454, zhu1_Ghi#S42298593) responsible for auxin transport were down-regulated. However, another three (zhu1_Ghi#S42308314, zhu1_Ghi#S42299563, zhu1_Ghi#S33832032) were up-regulated, while a *PIN3* homologous transcript (zhu1_Ghi#S42312756) was slightly up-regulated at 0–24 h and then down-regulated ( Additional file 9 Table S8-1). The expression profiles of selected genes were confirmed by qRT-PCR analysis (Figure 7B).

In addition, we found that several *Aux/IAA* genes were differentially expressed, though most Aux/IAA genes, except *IAA19*, *IAA14-1* and *AUX2-11-2* at some time points, were down-regulated during dedifferentiation, showed an extremely low pick at the EC stage and then were up-regulated during somatic embryo development (Figure 7C). Likewise, some ARF and SAUR genes were differentially expressed, with a complex expression profile. However, four genes (*ARF6-1*, *ARF9*, *NPH4* and *ARF6-2*) were up-regulated during somatic embryo development (Figure 7D). Some genes related to Aux/IAA degradation, such as *AFB2*, *TIR1*, *AXR1*, *RBX1*, *ASK2*, *ASK1*, *CUL1*, *RCE1*, *DCAF1* and *SGT1B* were also differentially expressed ( Additional file 9 Table S8-1).

Among the 86 genes, 58 genes were unique to the initial dedifferentiation process ( Additional file 9 Table S8-2). Transition from NECs to ECs triggered differential expression of 38 genes ( Additional file 9 Table S8-3), with 15 genes down-regulated and 23 genes up-regulated, while 35 genes were differentially expressed during somatic embryo development ( Additional file 9 Table S8-4).

### Comparison of DGE tag data with qRT-PCR

**Figure 8:**
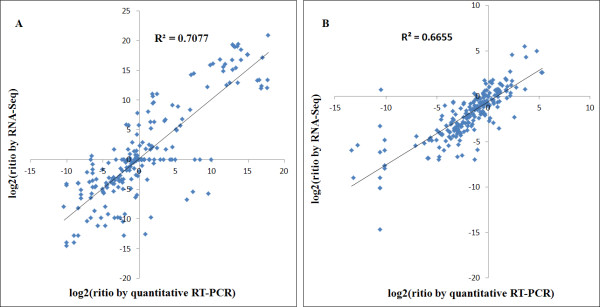
**Comparison of expression profile by RNA-Seq and qRT-PCR.** Comparison of expression profiles of 26 randomly selected genes by RNA-Seq and qRT-PCR showing different expression profiles with high or low expression levels at one or more time points during SE (**A**). Comparison of expression profiles of 26 genes by RNA-Seq and qRT-PCR related to auxin synthesis, transport and signalling (**B**)

To validate the expression profiles obtained by RNA-Seq, real-time RT-PCR was performed on 26 genes that showed different expression profiles during SE, with high or low expression levels at one or more time points. The Pearson correlation coefficient was calculated by SPSS to assess the correlation between different platforms. Overall, the qRT-PCR measurements were moderately correlated with the RNA-Seq results (Figure 8A, *R*^2^ = 0.7077, correlation was significant at the 0.01 level). An additional 26 genes related to auxin synthesis, transport and signalling were also validated by qRT-PCR, which showed a moderate correlation (Figure 8B, *R*^2^ = 0.6655, correlation was significant at the 0.01 level). For almost all genes tested, with the exception of a few genes at some time points, qRT-PCR analysis confirmed the direction of changes detected by DGE analysis.

Gene expression levels estimated by qRT-PCR at different time points/stages were also analysed. The correlation of RNA-Seq and qRT-PCR results during the dedifferentiation process (6 h and 24 h) was relatively low ( Additional file 10 Figure S2A, *R*^2^ = 0.2016 at 6 h, *R*^2^ = 0.1327 at 24 h). At 48 h, NEC and EC time points/stages showed a moderate correlation ( Additional file 10 Figure S2B, *R*^2^ = 0.5096 at 48 h, *R*^2^ = 0.5156 at NEC and *R*^2^ = 0.6365 at EC). In contrast, the correlations were higher at GE, TE and CE stages ( Additional file 10 Figure S2C, *R*^2^ = 0.8392 at GE, *R*^2^ = 0.8192 at TE and *R*^2^ = 0.8208 at CE). Although the samples collected early will be more affected by the sampling process and method, these results still suggest the applicability of RNA-Seq to cotton transcriptome analysis and confirm that it is an accurate and reliable way to find genes differentially expressed during dedifferentiation and redifferentiation.

## Discussion

### Applications and evaluation of DGE-based analysis with the reference database

Cotton is a major crop for fibre and oil production, and has been subject to the application of biotechnology for crop improvement. Cell culture and plant regeneration are the bases for cotton biotechnology through genetic transformation, and so understanding the molecular control of dedifferentiation and redifferentiation is key to manipulating the SE process. However, the large unsequenced genome size (approximately 2.5 gb), polyploid nature and lack of adequate gene model annotations have limited large-scale transcriptome analyses during cotton SE [39]. Previous studies on the molecular aspects used SSH and microarray [11,26] but provided limited information on the complex transcriptome dynamics during cotton SE. However, next-generation technologies, which can generate tens of thousands to tens of millions of sequence reads with exceptional reproducibility, provide new strategies to quantitatively analyse the functional complexity of transcriptomes, despite uncharacterized genome sequences [27,29,40].

Using RNA-Seq technology developed by Illumina and elite high efficient regeneration lines YZ1, we designed a protocol for analysing the transcriptome complexity of cotton SE. Although SE is usually divided into two stages, induction and expression [5], our morphological and histological observations indicate that it could usefully be divided into three different processes: dedifferentiation of somatic cells, transition from NECs to ECs and development of somatic embryos. Protoplasts undergo cellular dedifferentiation and initiate cell division within 48 to 72 h in tobacco and Arabidopsis [41,42]. Histological observations have shown that cotton somatic cells activate cellular dedifferentiation and division within 72 h, and often within 48 h [21]. We chose three different time points (6 h, 24 h and 48 h) for initial dedifferentiation sampling and typical NECs after 40 d of induction for late dedifferentiation. Different stages of somatic embryos were selected by distinct morphology observed after synchronization (Figure 1).

For genome sequence references that were unavailable, clean tags were mapped to two different EST reference databases after preprocessing the raw data. One reference database (Reference database 1) was cotton unigenes from NCBI that contains 20,671 unigene sequences, and the other (Reference database 2) was contigs assembly from multiple cotton genes from different databases which contain 65,386 sequences. The two reference databases were compared for efficiency based on several criteria. So many tags were missing using Reference database 2 for critical selection, that tags mapping to unique sequences were used for transcript identification ( Additional file 11 Table S9). However, validation by qRT-PCR of the expression profile for 26 differentially expressed genes derived by using RNA-Seq technology showed that genes mapped based on the two reference databases exhibited a similar correlation (*R*^2^ = 0.7077 for Reference database 1, and *R*^2^ = 0.7073 for Reference database 2) ( Additional file 12 Figure S3). As a result, we selected the cotton unigenes from NCBI (Reference database 1) as our reference database for further analysis.

Up to 50.7% (15,339) of the sequences in our reference database could be unambiguously identified by a unique tag. However, a relatively low number of the tags (43.18%) could be assigned to genes and used for gene expression profiling. This might be partly explained by the fact that most of the sequences in the database were not generated from embryogenesis development. More sequences and annotation for dedifferentiation and redifferentiation in cotton have to be explored to illuminate the large amount of unknown tags that remain. The extremely low abundance transcripts (TPM ≤ 20) were also filtered because of the possible of sequencing error. Among these, 5,076 differentially expressed genes were filtered with a cut-off of TPM ≥ 20, *P* ≤ 0.001 and the absolute value of log2Ratio ≥1 based on the FDR < 0.05 ( Additional file 3Table S2).

### Transcription regulation of somatic cells dedifferentiation and redifferentiation in cotton

Somatic cells within the plant contain all the genetic information necessary to create a complete and functional plant (with the exception of anuclear vascular cells). The induction of SE comprises the termination of one gene expression pattern in the explant tissue and replacement with an embryogenic gene expression programme [1]. The initiation of the embryogenic pathway, which is preceded by cellular dedifferentiation, is restricted only to certain responsive cells in the primary explant because the existing developmental information of somatic cells must be switched off or altered to make the somatic cells responsive for new signals [1,5]. Though we described the cotton SE as consisting of three different processes, it is very difficult to dissect the specific cellular events related to the overlapping phases of dedifferentiation, cell cycle reactivation and the acquisition of embryogenic competence.

The embryogenic processes are becoming better understood because of the identification of several genes such as transcription factors that play regulatory roles either in specific embryogenesis phases [43] or throughout the whole process [44]. In the present study, 466 TF mRNAs were differentially expressed over a wide range of abundances during SE. Among these, a subset of TF families were associated with functions in cell differentiation, embryogenic patterning and embryo maturation processes (Zinc finger, b-ZIP, bHLH, B3 and MYB), meristem maintenance or identity (NAC, YABBY, GRAS), while others had roles in hormone-mediated signalling by auxin (Aux/IAA, ARF) or ethylene (AP2/ERF). Zinc finger family proteins have been proven to be involved in cell differentiation and development processes in animals and plants [45,46]. *PEI1*, encoding a protein containing a Cys3-His zinc finger domain, is an embryo-specific transcription factor that plays an important role during Arabidopsis embryogenesis, functioning primarily in the apical domain of the embryo, which is required for the globular to heart-stage transition [46]. In the present study, genes encoding zinc finger family protein showed complex expression profiles ( Additional file 8Table S7), indicating that they have multiple functions during SE in cotton. In Arabidopsis, two bHLH proteins were required in embryogenic patterning for root formation in the embryo [47,48]. The diversity of expression profiles displayed by 78 bHLH homologues in the present study might suggest the complex regulation of SE by bHLH proteins in cotton ( Additional file 8Table S7).

B3 domain transcription factors in *Arabidopisis* (*LEC2FUS3* and *ABI3*) encode regulatory proteins involved in embryogenesis and induction of somatic embryo development [49,50]. Six B3 family transcription factor homologues were present with complex expression profiles: two (zhu1_Ghi#S33821461, zhu1_Ghi#S42277219) were down-regulated and one (zhu1_Ghi#S42340389) was up-regulated. Ectopic expression of *BABY BOOM* (*BBM*), a member of the AP2/ERF family in Arabidopsis primarily induces spontaneous somatic embryo formation from seedlings, although ectopic shoots and callus also develop at a lower frequency [51]. In our study, 26 AP2/ERF genes were differentially expressed during SE in cotton ( Additional file 8 Table S7). MYB and WRKY were transcription factors not only involved in response to biotic and/or abiotic stresses, but also regulated embryogenesis pathways [52,53]. As revealed in this study, most *MYB* genes were up-regulated during embryogenic initiation and showed a relatively low expression level during somatic embryo development, while most *MYB*-related genes were down-regulated during SE, indicating different functions of these genes during SE ( Additional file 8 Table S7). Further experiments are required to verify the physiological function and interaction between these factors and other genes during SE.

### Complex auxin signalling pathway during dedifferentiation and redifferentiation of cotton cells

The importance of PGRs during SE has been widely documented [4,54]. To understand better the hormonal regulation of SE, PGRs are added to a culture medium to induce somatic embryogenic process, and endogenous hormone concentrations of plant tissues are measured during morphogenesis or various developmental stages [55]. Auxin is considered to be a critical PGR in cell division and differentiation, as well as in the induction of SE. This regulation probably occurs by establishing auxin gradients during the induction phase of SE, essential for initiating dedifferentiation and cell division of already differentiated cells before they can express embryogenic competence [4]. Despite the absolute requirement for exogenous auxins to sustain growth in plant cells cultured *in vitro*, cultured plant cells produce substantial amounts of the native auxin, IAA. In carrot cells, exogenous auxin stimulates the accumulation of large amounts of endogenous IAA. Thus the application of exogenous auxin and subsequent endogenous auxin content are both determining factors during the induction phase [4,56]. For this gradient to be established, relatively high levels of IAA in the competent tissues may be necessary.

Most studies on hormone contents during induction of SE only evaluate NEC and EC cultures [32,57]. In the present study, the endogenous IAA concentrations were determined in the original somatic cells, phytohormone-induced dedifferentiation cells and embryogenic cells (Figure 2). *De novo* synthesis of IAA in these cells occurred under all the examined conditions. The endogenous levels of IAA declined to a half within 6 h and dropped to a quarter of the original values within 24–48 h following excision from seedlings (Figure 2). The kinetics of this decline in IAA levels was similar to the decline of IAA levels in wounded tobacco leaves by activation of the proteinase inhibitor gene system [58]. However, in IBA-treated soybean hypocotyls, IAA levels increased dramatically after wounding and reached a maximum after 24 h, with a decrease of the cationic peroxidase activity [59].

The mechanism responsible for the decline in IAA levels is not yet understood. The activation of some proteinase inhibitor genes in this study might be one possibility. The endogenous IAA could be influenced at one of several points, including its biosynthesis or degradation or the formation of amide or ester storage forms. Indeed, the decrease in IAA pools could even be influenced through IAA transport. Our data shed new light on these questions.

Our analysis revealed the dynamics of auxin levels during cotton SE (Figure 2). Previous studies have also shown that sharp changes in endogenous auxin levels may be one of the first steps leading to SE [31]. Redifferentiation was clearly correlated with a sharp increase in auxin responses in cotton cells, which provides direct evidence for the significance of an endogenous auxin pulse in the expression of cellular totipotency. It has also been noted that transition of the globular embryo to the heart-stage embryo and its further development requires either a low level of auxins or their complete absence [15]. Surprisingly, most RNA-Seq based auxin synthesis, transport, metabolism and signalling pathway genes were down-regulated during redifferentiation and somatic embryo development processes and showed a relatively low expression level in EC cultures ( Additional file 9 Table S8-1). However, transcript levels of genes related to auxin were changed during this process, indicating a possible role of this hormone in cotton SE.

The increase of IAA might be due to the increased synthesis and turnover of putative host auxin precursor in tissues. Although there are several IAA biosynthesis pathways in higher plants, tryptophan has long been regarded as the important one and its active metabolism and biosynthesis during embryogenesis have been highlighted [60]. A *TRP1* homologous transcript was differentially up-regulated during initial dedifferentiation at culture times of 6 h and 24 h (Figure 7A), with a consistent result in wheat [61], while *NIT4A* showed an opposite expression model (Figure 7A). Nitrilases can contribute to IAA homeostasis by hydrolyzing IAN to IAA in higher plants [62]. Conversion of Trp to IAA by enzymatic complex with nitrilase immunoreactivity *in vitro* was applied to plants [63]. Expression of maize nitrilase *ZmNIT2* is elevated in embryonic tissue [62]. In this study, *NIT4A* homologues were identified and were down-regulated during the whole process (Figure 7A), while qRT-PCR analysis of three additional nitrilase genes derived from cotton database gave a similar expression profile ( Additional file 13 Figure S4). In addition, the up-regulation of a *GH3.17* gene was observed in early dedifferentiation at 6 h of induction (Figure 7A). Several members of this family, including the *GH3* genes, were up-regulated at about 2–4 h in soybean hypocotyls exposed to auxin [64]. These enzymes encoded by members of the GH3 family are able to synthesize IAA amino acid conjugates. Two members of the Arabidopsis *GH3* gene family have been revealed to be overexpressed in dwarf mutants with reduced apical dominance combined with decreased free auxin levels [65,66]. Further, disruption of certain *GH3* genes confers hypersensitivity to specific forms of auxin conjugated by the encoded GH3 [67]. The characterized GH3 enzymes in this process might indicate that not only the level of free IAA but also the conjugated IAA is important during SE (Figure 7A).

Likewise, chemical and genetic studies have revealed that transport of auxin is complex and highly regulated for embryonic development [68]. Several Arabidopsis mutants are defective in proteins mediating polar auxin transport. AUX1, which mediates influx of IAA into cells, was localized asymmetrically in the plasma membrane of certain cell types, facilitating directional auxin transport [69]. Once IAA has entered a cell via AUX1, several factors regulate efflux. Three *AUX1* homologous transcripts (zhu1_Ghi#S33799879, zhu1_Ghi#S42308191 and zhu1_Ghi#S29994266) were down-regulated during cotton SE, while only *AUXI-LIKE* displayed high levels ( Additional file 9 Table S8-1). Like *AUX1PIN* is another gene family implicated in polar auxin transport, which is asymmetrically localized in the cell [68]. A *PIN3* transcript showed a high expression level during dedifferentiation but an extremely low level during the embryo development stage ( Additional file 9 Table S8-1). These results indicated the complex auxin flux during SE. More evidence is required, however, to prove a relationship between auxin transporters and auxin distribution during cotton SE.

Most of the auxin-inducible *Aux/IAA* transcripts, with the exception of one member (*IAA19*), had decreased expression levels during dedifferentiation and displayed extremely low levels at the EC stage but then increased during SE development (Figure 7C), showing a different pattern from endogenous auxin dynamics. Transcription changes of *Aux/IAA* genes after an auxin stimulus is likely to be mediated by ARF proteins via AuxREs in Aux/IAA promoter regions. ARFs can bind tandem repeat AuxRE sequences as homodimers, dimers with other ARFs or dimers with repressive Aux/IAA proteins [70]. Six auxin response factor–related genes were differentially expressed, with two showing the high expression levels (zhu1_Ghi#S42325122, zhu1_Ghi#S42278444 and zhu1_Ghi#S42310727) during the dedifferentiation process ( Additional file 9 Table S8-1). The up-regulation of some ARF transcripts might demonstrate an intimate connection between auxin responses and auxin levels during cotton SE (Figure 7D).

Genes connected to degradation of Aux/IAA proteins, such as the putative intracellular auxin receptor *TIR1*[71], was down-regulated during the process (Figure 7B), which was shown by two *TIR1* homologues (zhu1_Ghi#S37590130 and zhu1_Ghi#S33811942). *AXR1*, which is part of the auxin-induced Aux/IAA degradation machinery via the 26 S proteasome [72], was also differentially expressed ( Additional file 9 Table S8-1). Likewise, *ASK1* and *ASK2* are necessary for a proper auxin response, through interaction with the TIR1 F-box [73]. Two of four *ASK* homologous transcripts (zhu1_Ghi#S42334433 and zhu1_Ghi#S33808515) displayed relatively high expression levels, with *ASK2* down-regulated during the whole process and *ASK1* down-regulated during the initial dedifferentiation and then up-regulated during somatic embryo development ( Additional file 9 Table S8-1). These findings indicated the integration of the auxin signal pathways during cotton SE.

However, the expression profile of auxin-related genes revealed that the complex and redundant regulation of IAA abundance, transport and response allows an intricate system of auxin utilization that achieves a variety of purposes in SE. As a result, further study of these genes, from auxin biosynthesis to auxin metabolism, from regulated protein degradation to signal transduction cascades, from IAA abundance to auxin transport, is needed in cotton SE.

## Conclusions

Bioinformatics tools provide a powerful approach to identify changing patterns of gene expression during development. In the present study, through a combination of biochemical and histological approaches, we used RNA-Seq to investigate global gene expression patterns that regulate SE in cotton. This analysis represents a starting point for functional studies in SE, and further experimental research is required to expand on the findings obtained to define the molecular mechanisms underpinning the cellular patterning and biochemical differentiation of the embryogenic initiation and plant embryo development and the complex networks of interactions involved.

## Materials and methods

### Plant materials and culture conditions

Sterilized seeds of YZ1 (*Gossypium hirsutum* L*.*) were germinated on 1/2 MS (1/2 macro salts plus 15 g of glucose, pH 6.0) and cultured at 28 °C in the dark for 6 d. Hypocotyls were excised from germfree seedlings and cut into 5–7 mm segments. The explants were then cultured on MSB (MS medium plus B5 vitamins) medium supplemented with the combination of 1.0 mg/L IBA plus 0.1 mg/L kinetin. After 40 d of culture, all explants were transferred to fresh MSB medium for induction of embryogenic calli (ECs). The ECs were subcultured monthly on MSB medium, with KNO_3_ doubled but NH_4_NO_3_ removed, and supplemented with 3% (w/v) glucose, 0.25% (w/v) *Phytagel*, 0.5 mg/L IBA, 0.15 mg/L kinetin, 1.0 g/L glutamine and 0.5 g/L asparagines, for embryo maturation. All media were autoclaved at 121 °C for 15 min. Cultures were maintained in a room at 28 ± 2 °C under a 14-h photoperiod (irradiance of 135 μmol/m·s). Different stages of explants during initial cellular dedifferentiation (0 h, 6 h, 24 h, 48 h, 72 h, 5 d and 8 d), NECs (10 d, 15 d, 25 d and 40 d), ECs and somatic embryos [globular embryos (GEs), torpedo embryos (TEs) and cotyledon embryos (CEs)] were sampled as shown schematically in Figure 1.

A minimum of 50 explants (hypocotyls) from each type of medium were collected for the analysis of regeneration potential, including at least five replicates. Callus characteristics were recorded at 3 and 6 weeks for colour, texture, depressiveness in liquid and cell or callus sizes. Different stages of somatic embryos were synchronized by suspension culture.

### Histological analysis

To analyse the origin of ECs from hypocotyls in culture conditions, the samples were cut to small sections and fixed in FAA [10% formalin, 5% acetic acid, 50% ethanol (v/v)] at room temperature for at least 48 h before use. The dehydration and infiltration of the specimen were performed in ethanol and paraffin series as in a previous study [74]. After being embedded in paraffin, the samples were cut into semi-thin (4–8 μm) sections using a microtome (Leica RM2245, Germany) and stained with Safranin and Fast Green. Finally, the sections were observed under a microscope (Leica DM2500, Germany).

### Endogenous IAA extraction and quantification

The determination of endogenous IAA (free) was performed according to Liu et al. [75] with some modifications. Samples of different materials were immediately frozen in liquid nitrogen and stored at −70 °C until extracted. One hundred milligrams of each sample (fresh weight) were ground in liquid nitrogen, and then extracted overnight with 1 mL 80% cold aqueous methanol (containing 0.01% ascorbic acid as antioxidant) in darkness at 4 °C with shaking. Then the extract was centrifuged at 10,000 × *g* at 4 °C for 20 min. The supernatant was collected, and the residue was further extracted with an additional 0.4 mL of cold 80% aqueous methanol for 30 min and then centrifuged again; the supernatant was then mixed with the previous one. After evaporating to aqueous phase in N_2_, the extracts were dissolved in 0.3 mL of methanol and filtered through a 0.45 μm nylon membrane and then stored at −20 °C before measurement. Each sample had three replicates; IAA was then quantified with an Applied Biosystems 4000Q-TRAR HPLC-MS system (Applied 24 Biosystems, USA) with IAA (Sigma-Aldrich, St. Louis, MO) as the standard in an external standard method.

### RNA extraction, library construction and sequencing

Different stage of cotton materials during initial cellular dedifferentiation (0 h, 6 h, 24 h, and 48 h), NECs, ECS and somatic embryos (GEs, TEs, and CEs) were collected for RNA extraction as shown schematically in Figure 1. Total RNA was isolated from each sample by using a modified guanidine thiocyanate method [76]. Twenty micrograms of total RNA was sent to Beijing Genomics Institute (Shenzhen) where the libraries were constructed and sequenced using Illumina’s Genome Analyzer. RNA quality and quantity were determined by using a Nanodrop 2000 instrument (Thermo Scientific) and a Bioanalyzer Chip RNA7500 series II (Agilent). Total RNA (1–2 μg) was fractionated using oligo-dT magnetic beads to yield polyA mRNA. mRNA bound to the beads was then used as a template for first strand cDNA synthesis primed by oligo-dT and the second strand cDNA was consequently synthesized using random primers. The 3′ tag DGE libraries were constructed from different materials essentially as described in previous studies [28]. Briefly, the cDNA was digested with *Nla*III, which recognizes the CATG site, and then ligated with the Illumina GEX *Nla*III Adapter 1 containing the recognition site of *Mme*I. Digestion with *Mme*I yielded the adapter tag linked to 21 bp of cDNA including 4 bp of the *Nla*III recognition site. After digestion by *Mme*I, the transcripts were ligated with the GEX Adapter 2. With the sequencing primers designed based on the two adaptors, the sequence of the 21 bp representing each transcript can be determined via a series of enzymatic reactions on the microbeads. The derived reliable sequence was termed signature herein. The abundance of each signature was normalized to one million for the purpose of comparison between samples.

### Analysis of DGE tags and bioinformatics

Sequencing output raw data were first filtered to remove adaptor tags, low quality sequences (tags with unknown sequences ‘N’) and tags with a copy number of 1 (probably sequencing error). For annotation, all tags were mapped to the reference sequences (cotton unigenes from NCBI) and allowed no more than one nucleotide mismatch. All the tags mapped to reference sequences from multiple genes were filtered and the remaining tags were designed as unambiguous tags. For gene expression analysis, the number of expressed tags was calculated and then normalized to TPM (number of transcripts per million clean tags), a normalized measure of read density that allows transcript levels to be compared both within and between samples [33]. Because ERANGE distributes multi reads at similar loci in proportion to the number of unique reads recorded, we included the analysis of both unique reads and reads that occur up to 20 times to avoid undercounting genes that have closely related paralogs [77]. To minimize false positives and negatives, we estimated that statistical analysis was reliable when applied to genes showing a TPM ≥ 20 in at least one of these stages. It should be noted that the statistical significance was based on expected sampling distributions. Due to the use of a single biological replicate for each time point, these high levels of significance may not reflect biological differences caused by development but may instead reflect other differences among the samples. To obtain statistical confirmation of the differences in gene expression among the developmental stages, we then compared the TPM-derived read count using a threshold value of *P* ≤ 0.001 and the absolute value of log2Ratio ≥1 based on the FDR < 0.05.

### Annotation and functional classification

To assign putative functions to differentially expressed genes, BLAST search was done against both GenBank non-redundant protein and TAIR9 protein sequences of *Arabidopsis thaliana* (http://www.arabidopsis.org/) using BLASTx program (E-value ≥ 10^−5^) [24]. To identify putative transcription factors, the BLASTx was done against an publicly available Arabidopsis TF databases [Database of Arabidopsis Transcription Factors (DATF)] [78], Plant Transcription Factor Database (PlnTFDB) [79] and cotton Transcription Factor (http://planttfdb.cbi.pku.edu.cn:9010/web/index.php?sp=gh). The analysis of biological processes/pathways was carried out using the KEGG [34] Automatic Annotation Server with the SBH option checked and plant gene datasets selected. Functional annotation by GO terms (http://www.geneontology.org) was analysed using Blast2GO software based on BLASTx against NCBI non-redundant (nr) protein database. Furthermore, expression profile of differentially expressed genes was accomplished by hierarchical clustering with k-means method using Pearson’s correlation distance based on their expression modulation by Genesis [38].

### qRT-PCR validation

qRT-PCR was carried out to estimate the validity of RNA-Seq technology for expression profile analysis. Gene-specific primers ( Additional file 14  Table S10) were designed according to the cDNAs sequences with Primer Premire 5 (http://www.premierbiosoft.com/crm/jsp/com/pbi/crm/clientside/ProductList.jsp) and synthesized commercially (*Genscript* Bioscience, Nanjing). First-strand cDNA was generated from 3 μg RNA samples by using Superscript III RT (Invitrogen), and the products were adjusted to initial RNA concentration of 2 ng/μL for qRT-PCR. The cDNA templates were diluted 500 times prior to amplification. qRT-PCR was performed in 20 μL reactions in triplicate on an ABI Prism 7000 Real-time PCR system (Foster City, CA, USA) according to our previous study [80] using 5 μL of first-strand cDNAs as templates, 10 μL of 2 × SYBR Green PCR Master Mix (Applied Biosystems), 0.5 μL of each 20 μM forward and reverse gene-specific primers and 4 μL of PCR-grade water into 96-well plates. As a control, the polyubiquitin transcripts were used as internal standards. Thermal cycling conditions was performed with an initial denaturation step of 1 min at 95°C, followed by 40 cycles of 15 s at 95°C, 58 °C for 15 s and 72 °C for 45 s. Following amplification, a dissociation stage was carried out to detect any complex products. Data analysis was performed with RQ Manager Software (Applied Bioscience). Relative quantitation of gene expression was calculated and normalized using cotton ubiquitin gene as an internal standard and the relative expression ratio value was calculated for development time points relative to the first sampling time point.

Sequence data for this article have been deposited in the National Center for Biotechnology Information Gene Expression Omnibus, and are accessible through GEO Series accession number GSE38209.

## Authors' contributions

XY designed the experiment and carried out the experiment with FJ, YZ and JX. XY drafted and wrote the manuscript. XZ designed the research and revised the manuscript. XY and DY performed the statistical analysis of the data. All authors read and approved the final manuscript.

## Supplementary Material

Additional file 1 **Figure S1. Sequencing saturation analysis of different libraries.** Newly emerging distinct tags were gradually reduced as the total number of sequence tags rose. The library capacity approached saturation when the number of sequencing tags reached 2–2.5 million. (TIFF 6197 kb) Click here for file

Additional file 2 **Table S1. Statistics of DGE sequencing.** (XLS 30 kb) Click here for file

Additional file 3 **Table S2. The summary of the tag information and gene expression level.** 15,339 sequences in the reference database could be unambiguously identified by unique tags. (XLS 5674 kb) Click here for file

Additional file 4 **Table S3. The tag information, gene expression level, BLASTx against nr and TAIR9 protein sequence with E-value ≤10**^**−5**^** for 5,076 differentially expressed genes.** (XLS 3454 kb) Click here for file

Additional file 5 **Table S4. 2,118 sequences out of 5,076 differentially expressed genes were assigned to 256 biochemical pathways by KEGG pathways analysis.** (XLS 58 kb) Click here for file

Additional file 6 **Table S5. The GO term for the 5,070 differentially expressed genes.** (XLS 427 kb) Click here for file

Additional file 7 **Table S6. Expression data (log2-transformed) of each type genes.** Table S6-1. Expression data (log2-transformed) of genes in Type I. Table S6-2. Expression data (log2-transformed) of genes in Type II. Table S6-3. Expression data (log2-transformed) of genes in Type III. Table S6-4. Expression data (log2-transformed) of genes in Type IV. Table S6-5. Expression data (log2-transformed) of genes in Type V. (XLSX 343 kb) Click here for file

Additional file 8 **Table S7. List and categories of putative transcription factors.** Table S7-1. Transcription factors differentially expressed throughout the whole process. Table S7-2. Transcription factors differentially expressed during the initial dedifferentiation. Table S7-3. Transcription factors differentially expressed during the transition from NECs to ECs. Table S7-4. Transcription factors differentially expressed during somatic embryo development. (XLS 487 kb) Click here for file

Additional file 9 **Table S8. The tag information and gene expression of level of auxin-related genes differentially expressed during the process.** Table S8-1. The tag information and gene expression level of auxin-related genes differentially expressed throughout the whole process. Table S8-2. The tag information and gene expression level of auxin-related genes differentially expressed during the initial dedifferentiation. Table S8-3. The tag information and gene expression level of auxin-related genes differentially expressed during the transition from NECs to ECs. Table S8-4. The tag information and gene expression level of auxin-related genes differentially expressed during somatic embryo development. (XLS 183 kb) Click here for file

Additional file 10 **Figure S2. The correlation of expression levels revealed by RNA-Seq and qRT-PCR.** The correlation of RNA-Seq and qRT-PCR during the dedifferentiation process (6 h and 24 h) was relatively low (A), while 48 h NEC and EC time points/stages showed moderate correlation (B). The correlations were higher in the GE, TE and CE stages (C). (TIFF 3140 kb) Click here for file

Additional file 11 **Table S9. Comparison of two reference databases.** So many tags were missing using Reference database 2 for critical selection, that tags mapping to unique sequences were used for transcript identification. (XLS 24 kb) Click here for file

Additional file 12 **Figure S3. The correlation expression levels by RNA-Seq and qRT-PCR using two reference databases.** The correlation of expression profiles from RNA-Seq and qRT-PCR of 26 randomly selected differentially expressed genes mapped using Reference database 1 (A) and Reference database 2 (B). (TIFF 2790 kb) Click here for file

Additional file 13 **Figure S4. qRT-PCR analysis of four nitrilases genes derived from cotton database gave the similar expression profile.** (TIFF 1361 kb) Click here for file

Additional file 14 **Table S10. Gene-specific primers used for qRT-PCR.** Table S10-1. Primers of cotton ubiquitin gene (as internal standard) and 26 genes that showed different expression profiles during SE used for qRT-PCR. Table S10-2. Primers of 26 auxin-related genes used for qRT-PCR. (XLS 36 kb) Click here for file

## References

[B1] Yang XY Zhang XL Regulation of somatic embryogenesis in higher plants Crit Rev Plant Sci 2010 29 36 57 10.1080/07352680903436291

[B2] Quiroz-Figueroa FR Rojas-Herrera R Galaz-Avalos RM Loyola-Vargas VM Embryo production through somatic embryogenesis can be used to study cell differentiation in plants Plant Cell, Tiss Organ Cult 2006 86 285 301 10.1007/s11240-006-9139-6

[B3] Domoki M Györgyey J Bíró J Pasternak TP Zvara Á Bottka S Puskás LG Dudits D Fehér A Identification and characterization of genes associated with the induction of embryogenic competence in leaf-protoplast-derived alfalfa cells Biochim Biophys Acta 2006 1759 543 551 10.1016/j.bbaexp.2006.11.005 17182124

[B4] Jiménez VM Involvement of plant hormones and plant growth regulators on in vitro somatic embryogenesis Plant Growth Regul 2005 47 91 110 10.1007/s10725-005-3478-x

[B5] Fehér A Pasternak TP Dudits D Transition of somatic plant cells to an embryogenic state Plant Cell, Tiss Organ Cult 2003 74 201 228 10.1023/A:1024033216561

[B6] Marcel AJ Toonen TH Ed DL S Verhoeven HA van Kammen A DeVries SC Description of somatic-embryo-forming single cells in carrot suspension cultures employing video cell tracking Planta 1994 194 8

[B7] Thibaud-Nissen F Clustering of microarray data reveals transcript patterns associated with somatic embryogenesis in soybean Plant Physiol 2003 132 118 136 10.1104/pp.103.019968 12746518 PMC166958

[B8] Kurczyńska EU Gaj MD Ujczak A Mazur E Histological analysis of direct somatic embryogenesis in Arabidopsis thaliana (L.) Heynh Planta 2007 226 619 628 10.1007/s00425-007-0510-6 17406890

[B9] Sung ZR Okimoto R Coordinate gene expression during somatic embryogenesis in carrots Proc Natl Acad Sci USA 1983 80 2661 2665 10.1073/pnas.80.9.2661 16593311 PMC393887

[B10] Sato S Toya T Kawahara R Whittier RF Fukuda H Komamine A Isolation of a carrot gene expressed specifically during early-stage somatic embryogenesis Plant Mol Biol 1995 28 39 46 10.1007/BF00042036 7787186

[B11] Zeng FC Zhang XL Zhu LF Tu L Guo XP Nie YC Isolation and characterization of genes associated to cotton somatic embryogenesis by suppression subtractive hybridization and macroarray Plant Mol Biol 2006 60 167 183 10.1007/s11103-005-3381-x 16429258

[B12] Su YH Zhao XY Liu YB Zhang CL O’Neill SD Zhang XS Auxin-induced WUS expression is essential for embryonic stem cell renewal during somatic embryogenesis in Arabidopsis Plant J 2009 59 448 460 10.1111/j.1365-313X.2009.03880.x 19453451 PMC2788036

[B13] Mantiri FR Kurdyukov S Lohar DP Sharopova N Saeed NA Wang X-D VandenBosch KA Rose RJ The transcription factor MtSERF1 of the ERF subfamily identified by transcriptional profiling is required for somatic embryogenesis induced by auxin plus cytokinin in Medicago truncatula Plant Physiol 2008 146 1622 1636 10.1104/pp.107.110379 18235037 PMC2287338

[B14] Sharma S Millam S Hedley P McNicol J Bryan G Molecular regulation of somatic embryogenesis in potato: an auxin led perspective Plant Mol Biol 2008 68 185 201 10.1007/s11103-008-9360-2 18553172

[B15] Suprasanna P Bapat V Mujib A, Šamaj J Differential gene expression during somatic embryogenesis Somatic Embryogenesis. Volume 2 2006 Springer, Berlin/Heidelberg 305 320

[B16] James C Global Status of Commercialized Biotech/GM Crops: 2010.ISAAA Brief No. 42 2010 ISAAA, Ithaca, NY

[B17] Wilkins T Rajasekaran K Anderson DM Cotton biotechnology Crit Rev Plant Sci 2000 19 511 550 10.1016/S0735-2689(01)80007-1

[B18] Kumria R Sunnichan VG Das DK Gupta SK Reddy VS Bhatnagar RK Leelavathi S High-frequency somatic embryo production and maturation into normal plants in cotton (Gossypium hirsutum) through metabolic stress Plant Cell Rep 2003 21 635 639 12789412 10.1007/s00299-002-0554-9

[B19] Trolinder NL Goodin JR Somatic embryogenesis and plant regeneration in cotton (Gossypium hirsutum L.) Plant Cell Rep 1987 6 231 234 10.1007/BF00268487 24248660

[B20] Jin SX Zhang XL Nie YC Guo XP Liang SG Zhu HG Identification of a novel elite genotype for in vitro culture and genetic transformation of cotton Biol Plantarum 2006 50 519 524 10.1007/s10535-006-0082-5

[B21] Zhu HG Tu LL Jin SX Xu L Tan JF Deng FL Zhang XL Analysis of genes differentially expressed during initial cellular dedifferentiation in cotton Chinese Sci Bull 2008 53 3666 3676 10.1007/s11434-008-0468-1

[B22] Hecht V Vielle-Calzada JP Hartog MV Schmidt EDL Boutilier K Grossniklaus U de Vries SC The Arabidopsis somatic embryogenesis receptor kinase 1 gene is expressed in developing ovules and embryos and enhances embryogenic competence in culture Plant Physiol 2001 127 803 816 10.1104/pp.010324 11706164 PMC129253

[B23] Grabowska A Wisniewska A Tagashira N Malepszy S Filipecki M Characterization of CsSEF1 gene encoding putative CCCH-type zinc finger protein expressed during cucumber somatic embryogenesis J Plant Physiol 2009 166 310 323 10.1016/j.jplph.2008.06.005 18778873

[B24] Hu LS Yang XY Yuan DJ Zeng FC Zhang XL GhHmgB3 deficiency deregulates proliferation and differentiation of cells during somatic embryogenesis in cotton Plant Biotechnol J 2011 9 1038 1048 10.1111/j.1467-7652.2011.00617.x 21554528

[B25] Leng CX Li FG Chen GY Liu CL cDNA-AFLP analysis of somatic embryogenesis at early stage in TM-1 (Gossypium hirsutum L.) Xibei Zhiwu Xuebao 2007 27 233 237

[B26] Wu XM Li FG Zhang CJ Liu CL Zhang XY Differential gene expression of cotton cultivar CCRI24 during somatic embryogenesis J Plant Physiol 2009 166 1275 1283 10.1016/j.jplph.2009.01.012 19328593

[B27] Zheng ZL Advani A Melefors Ö Glavas S Nordström H Ye W Engstrand L Andersson AF Titration-free massively parallel pyrosequencing using trace amounts of starting material Nucleic Acids Res 2010 38 e137 10.1093/nar/gkq332 20435675 PMC2910068

[B28] Wang XW Luan JB Li JM Bao YY Zhang CX Liu SS De novo characterization of a whitefly transcriptome and analysis of its gene expression during development BMC Genomics 2010 11 400 10.1186/1471-2164-11-400 20573269 PMC2898760

[B29] Tang FC Barbacioru C Wang YZ Nordman E Lee C Xu NL Wang XH Bodeau J Tuch BB Siddiqui A mRNA-Seq whole-transcriptome analysis of a single cell Nat Methods 2009 6 377 382 10.1038/nmeth.1315 19349980

[B30] Xiang D Venglat P Tibiche C Yang H Risseeuw E Cao Y Babic V Cloutier M Keller W Wang E Genome-wide analysis reveals gene expression and metabolic network dynamics during embryo development in Arabidopsis Plant Physiol 2011 156 346 356 10.1104/pp.110.171702 21402797 PMC3091058

[B31] Thomas C Bronner R Molinier J Prinsen E van Onckelen H Hahne G Immuno-cytochemical localization of indole-3-acetic acid during induction of somatic embryogenesis in cultured sunflower embryos Planta 2002 215 577 583 10.1007/s00425-002-0791-8 12172840

[B32] Zeng FC Zhang XL Jin SX Cheng L Liang SG Hu LS Guo XP Nie YC Cao JL Chromatin reorganization and endogenous auxin/cytokinin dynamic activity during somatic embryogenesis of cultured cotton cell Plant Cell, Tiss Organ Cult 2007 90 63 70 10.1007/s11240-007-9253-0

[B33] Morrissy AS Morin RD Delaney A Zeng T McDonald H Jones S Zhao Y Hirst M Marra MA Next-generation tag sequencing for cancer gene expression profiling Genome Res 2009 19 1825 1835 10.1101/gr.094482.109 19541910 PMC2765282

[B34] Kanehisa M Goto S KEGG: Kyoto Encyclopedia of Genes and Genomes Nucleic Acids Res 2000 28 27 30 10.1093/nar/28.1.27 10592173 PMC102409

[B35] von Arnold S Sabala I Bozhkov P Dyachok J Filonova L Developmental pathways of somatic embryogenesis Plant Cell, Tiss Organ Cult 2002 69 233 249 10.1023/A:1015673200621

[B36] Botstein D Cherry JM Ashburner M Ball CA Blake JA Butler H Davis AP Dolinski K Dwight SS Eppig JT Gene Ontology: tool for the unification of biology Nat Genet 2000 25 25 29 10.1038/75556 10802651 PMC3037419

[B37] Gotz S Garcia-Gomez JM Terol J Williams TD Nagaraj SH Nueda MJ Robles M Talon M Dopazo J Conesa A High-throughput functional annotation and data mining with the Blast2GO suite Nucleic Acids Res 2008 36 3420 3435 10.1093/nar/gkn176 18445632 PMC2425479

[B38] Zenoni S Ferrarini A Giacomelli E Xumerle L Fasoli M Malerba G Bellin D Pezzotti M Delledonne M Characterization of transcriptional complexity during berry development in Vitis vinifera using RNA-Seq Plant Physiol 2010 152 1787 1795 10.1104/pp.109.149716 20118272 PMC2850006

[B39] Chen ZJ Scheffler BE Dennis E Triplett BA Zhang T Guo W Chen X Stelly DM Rabinowicz PD Town CD Toward sequencing cotton (Gossypium) genomes Plant Physiol 2007 145 1303 1310 10.1104/pp.107.107672 18056866 PMC2151711

[B40] Marioni J Mason C Mane S Stephens M Gilad Y RNA-seq: an assessment of technical reproducibility and comparison with gene expression arrays Genome Res 2008 18 1509 1517 10.1101/gr.079558.108 18550803 PMC2527709

[B41] Zhao J Morozova N Williams L Libs L Avivi Y Graf G Two phases of chromatin decondensation during dedifferentiation of plant cells J Biol Chem 2001 276 22772 22778 10.1074/jbc.M101756200 11274191

[B42] Dovzhenko A Dal Bosco C Meurer J Koop HU Efficient regeneration from cotyledon protoplasts in Arabidopsis thaliana Protoplasma 2003 222 107 111 10.1007/s00709-003-0011-9 14513316

[B43] Jenik PD Gillmor CS Lukowitz W Embryonic patterning in Arabidopsis thaliana Annu Rev Cell Dev Bi 2007 23 207 236 10.1146/annurev.cellbio.22.011105.102609 17539754

[B44] Park S Harada JJ Arabidopsis embryogenesis Method Mol Biol 2008 427 3 16 10.1007/978-1-59745-273-1_1 18369993

[B45] Huang CX Jia YC Yang SL Chen B Sun HW Shen F Wang YZ Characterization of ZNF23, a KRAB-containing protein that is downregulated in human cancers and inhibits cell cycle progression Exp Cell Res 2007 313 254 263 10.1016/j.yexcr.2006.10.009 17137575

[B46] Li ZS Thomas TL PEI1, an embryo-specific zinc finger protein gene required for heart-stage embryo formation in Arabidopsis Plant Cell 1998 10 383 398 9501112 10.1105/tpc.10.3.383 PMC143998

[B47] Schlereth A Moller B Liu W Kientz M Flipse J Rademacher EH Schmid M Jurgens G Weijers D MONOPTEROS controls embryonic root initiation by regulating a mobile transcription factor Nature 2010 464 913 916 10.1038/nature08836 20220754

[B48] Tsukagoshi H Busch W Benfey PN Transcriptional regulation of ROS controls transition from proliferation to differentiation in the root Cell 2010 143 606 616 10.1016/j.cell.2010.10.020 21074051

[B49] Gaj MD Zhang S Harada JJ Lemaux PG Leafy cotyledon genes are essential for induction of somatic embryogenesis of Arabidopsis Planta 2005 222 977 988 10.1007/s00425-005-0041-y 16034595

[B50] Braybrook S Harada J LECs go crazy in embryo development Trends Plant Sci 2008 13 624 630 10.1016/j.tplants.2008.09.008 19010711

[B51] Boutilier K Offringa R Sharma VK Kieft H Ouellet T Zhang L Hattori J Liu C-M van Lammeren AAM Miki BLA Ectopic expression of BABY BOOM triggers a conversion from vegetative to embryonic growth Plant Cell 2002 14 1737 1749 10.1105/tpc.001941 12172019 PMC151462

[B52] Lagacé M Matton D Characterization of a WRKY transcription factor expressed in late torpedo-stage embryos of Solanum chacoense Planta 2004 219 185 189 10.1007/s00425-004-1253-2 15045588

[B53] Wang XC Niu QW Teng C Li C Mu J Chua NH Zuo JR Overexpression of PGA37/MYB118 and MYB115 promotes vegetative-to-embryonic transition in Arabidopsis Cell Res 2009 19 224 235 10.1038/cr.2008.276 18695688

[B54] Feher A Pasternak TP Dudits D Transition of somatic plant cells to an embryogenic state Plant Cell, Tiss Organ Cult 2003 74 201 228 10.1023/A:1024033216561

[B55] Jiménez VM Guevara E Herrera J Bangerth F Endogenous hormone levels in habituated nucellar Citrus callus during the initial stages of regeneration Plant Cell Rep 2001 20 92 100 10.1007/s002990000280 30759920

[B56] Michalczuk L Ribnicky DM Cooke TJ Cohen JD Regulation of indole-3-acetic acid biosynthetic pathways in carrot cell cultures Plant Physiol 1992 100 1346 1353 10.1104/pp.100.3.1346 16653127 PMC1075788

[B57] Jiménez VM Bangerth F Endogenous hormone concentrations and embryogenic callus development in wheat Plant Cell, Tiss Organ Cult 2001 67 37 46 10.1023/A:1011671310451

[B58] Thornburg RW Li X Wounding Nicotiana tabacum leaves causes a decline in endogenous indole-3-acetic acid Plant Physiol 1991 96 802 805 10.1104/pp.96.3.802 16668256 PMC1080846

[B59] Chao IL Cho CL Chen LM Liu ZH Effect of indole-3-butyric acid on the endogenous indole-3-acetic acid and lignin contents in soybean hypocotyl during adventitious root formation J Plant Physiol 2001 158 1257 1262 10.1078/0176-1617-00500

[B60] Siriwaradna S Nabors MW Tryptophan enhancement of somatic embryogenesis in rice Plant Physiol 1983 73 142 146 10.1104/pp.73.1.142 16663163 PMC1066423

[B61] Chen JY Ma PA Zhao YD Zhu X-P Cui Y Zhang YM Chen XJ Expression of auxin-related genes during dedifferentiation of mature embryo in wheat Acta Agronomica Sinica 2009 35 1798 1805

[B62] Park WJ Kriechbaumer V Müller A Piotrowski M Meeley RB Gierl A Glawischnig E The nitrilase ZmNIT2 converts indole-3-acetonitrile to indole-3-acetic acid Plant Physiol 2003 133 794 802 10.1104/pp.103.026609 12972653 PMC219053

[B63] Normanly J Grisafi P Fink GR Bartel B Arabidopsis mutants resistant to the auxin effects of indole-3-acetonitrile are defective in the nitrilase encoded by the NIT1 gene Plant Cell 1997 9 1781 1790 9368415 10.1105/tpc.9.10.1781 PMC157021

[B64] Hagen G Kleinschmidt A Guilfoyle T Auxin-regulated gene expression in intact soybean hypocotyl and excised hypocotyl sections Planta 1984 162 147 153 10.1007/BF00410211 24254049

[B65] Nakazawa M Yabe N Ichikawa T Yamamoto YY Yoshizumi T Hasunuma K Matsui M DFL1, an auxin-responsive GH3 gene homologue, negatively regulates shoot cell elongation and lateral root formation, and positively regulates the light response of hypocotyl length Plant J 2001 25 213 221 10.1046/j.1365-313x.2001.00957.x 11169197

[B66] Takase T Nakazawa M Ishikawa A Kawashima M Ichikawa T Takahashi N Shimada H Manabe K Matsui M ydk1-D, an auxin-responsive GH3 mutant that is involved in hypocotyl and root elongation Plant J 2004 37 471 483 10.1046/j.1365-313X.2003.01973.x 14756757

[B67] Staswick PE Serban B Rowe M Tiryaki I Maldonado MT Maldonado MC Suza W Characterization of an Arabidopsis enzyme family that conjugates amino acids to indole-3-acetic acid Plant Cell 2005 17 616 627 10.1105/tpc.104.026690 15659623 PMC548830

[B68] Friml J Vieten A Sauer M Weijers D Schwarz H Hamann T Offringa R Jurgens G Efflux-dependent auxin gradients establish the apical-basal axis of Arabidopsis Nature 2003 426 147 153 10.1038/nature02085 14614497

[B69] Swarup R Kargul J Marchant A Zadik D Rahman A Mills R Yemm A May S Williams L Millner P Structure function analysis of the presumptive Arabidopsis auxin permease AUX1 Plant Cell 2004 16 3069 3083 10.1105/tpc.104.024737 15486104 PMC527199

[B70] Kim J Harter K Theologis A Protein–protein interactions among the Aux/IAA proteins Proc Natl Acad Sci USA 1997 94 11786 11791 10.1073/pnas.94.22.11786 9342315 PMC23574

[B71] Kepinski S Leyser O The Arabidopsis F-box protein TIR1 is an auxin receptor Nature 2005 435 446 451 10.1038/nature03542 15917798

[B72] Leyser HMO Lincoln CA Timpte C Lammer D Turner J Estelle M Arabidopsis auxin-resistance gene AXR1 encodes a protein related to ubiquitin-activating enzyme E1 Nature 1993 364 161 164 10.1038/364161a0 8321287

[B73] Gray WM Kepinski S Rouse D Leyser O Estelle M Auxin regulates SCFTIR1-dependent degradation of AUX/IAA proteins Nature 2001 414 271 276 10.1038/35104500 11713520

[B74] Yang XY Zhang XL Fu L-L Min L Liu GZ Multiple shoots induction in wild cotton (Gossypium bickii) through organogenesis and the analysis of genetic homogeneity of the regenerated plants Biologia 2010 65 496 503 10.2478/s11756-010-0037-3

[B75] Liu BF Zhong XH Lu YT Analysis of plant hormones in tobacco flowers by micellar electrokinetic capillary chromatography coupled with on-line large volume sample stacking J Chromatogr A 2002 945 257 265 10.1016/S0021-9673(01)01503-5 11860141

[B76] Zhu LF Tu LL Zeng FC Liu DQ Zhang XL An improved simple protocol for isolation of high quality RNA from Gossypium spp. suitable for cDNA library construction Acta Agronomica Sinica 2005 31 1657 1659

[B77] Marioni JC Mason CE Mane SM Stephens M Gilad Y RNA-seq: An assessment of technical reproducibility and comparison with gene expression arrays Genome Res 2008 18 1509 1517 10.1101/gr.079558.108 18550803 PMC2527709

[B78] Guo A He K Liu D Bai S Gu X Wei L Luo J DATF: a database of Arabidopsis transcription factors Bioinformatics 2005 21 2568 2569 10.1093/bioinformatics/bti334 15731212

[B79] Guo AY Chen X Gao G Zhang H Zhu QH Liu XC Zhong YF Gu XC He K Luo JC PlantTFDB: a comprehensive plant transcription factor database Nucleic Acids Res 2008 36 suppl 1 D966 D969 17933783 10.1093/nar/gkm841 PMC2238823

[B80] Yang XY Tu LL Zhu LF Fu LL Min L Zhang XL Expression profile analysis of genes involved in cell wall regeneration during protoplast culture in cotton by suppression subtractive hybridization and macroarray 2008 59 3661 3674 10.1093/jxb/ern214 PMC2561149 18775953

