# Supplementary figures and images for: Transcript profiling reveals complex auxin signalling pathway and transcription regulation involved in dedifferentiation and redifferentiation during somatic embryogenesis in cotton
Source: BMC Plant Biol. 2012 Jul 20;12:110. doi: 10.1186/1471-2229-12-110 (PMC3483692; doi:10.1186/1471-2229-12-110)

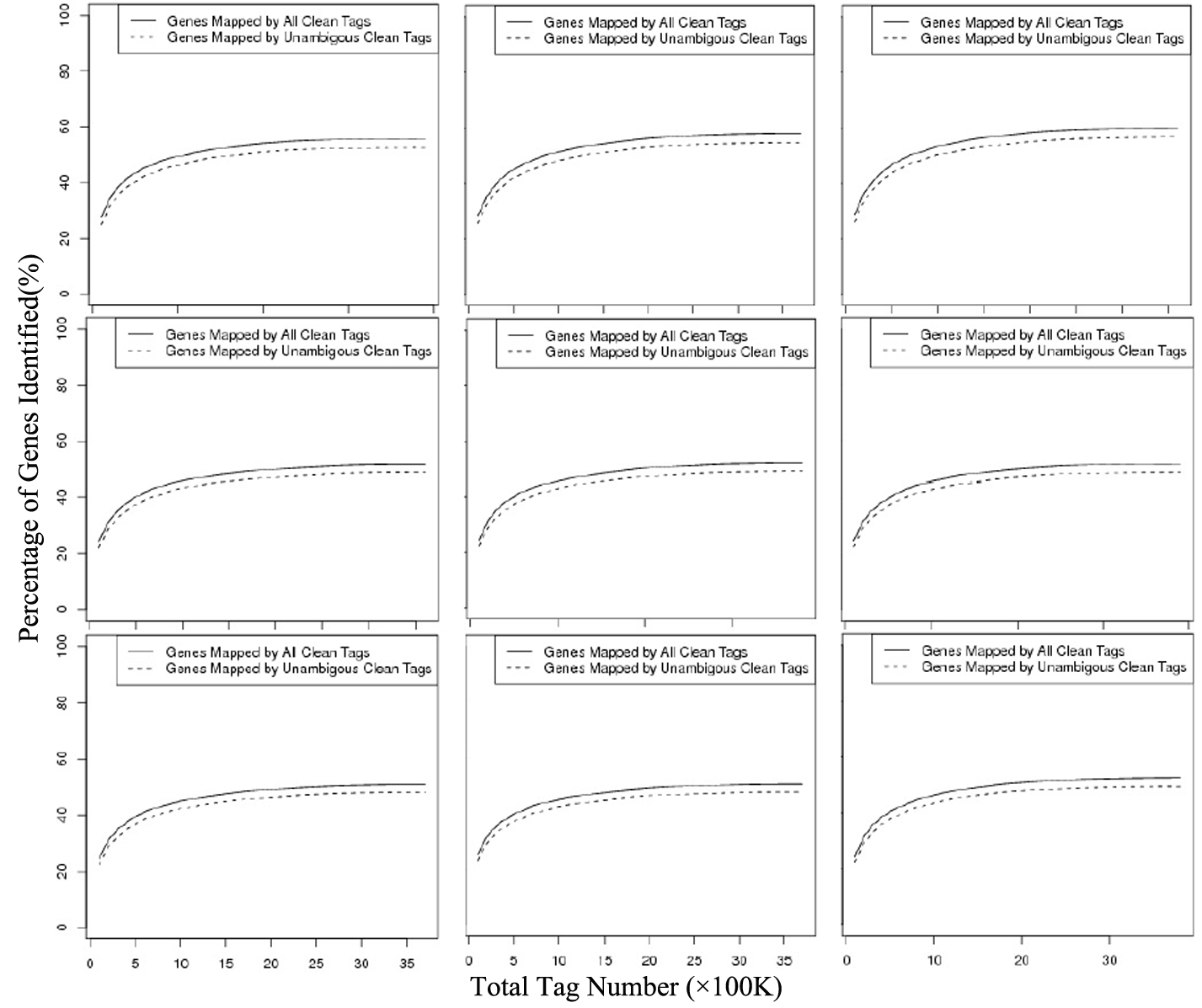

Supplement: Additional file 1 — Figure S1. Sequencing saturation analysis of different libraries. Newly emerging distinct tags were gradually reduced as the total number of sequence tags rose. The library capacity approached saturation when the number of sequencing tags reached 2–2.5 million. (TIFF 6197 kb) [file 1471-2229-12-110-S1.tiff]

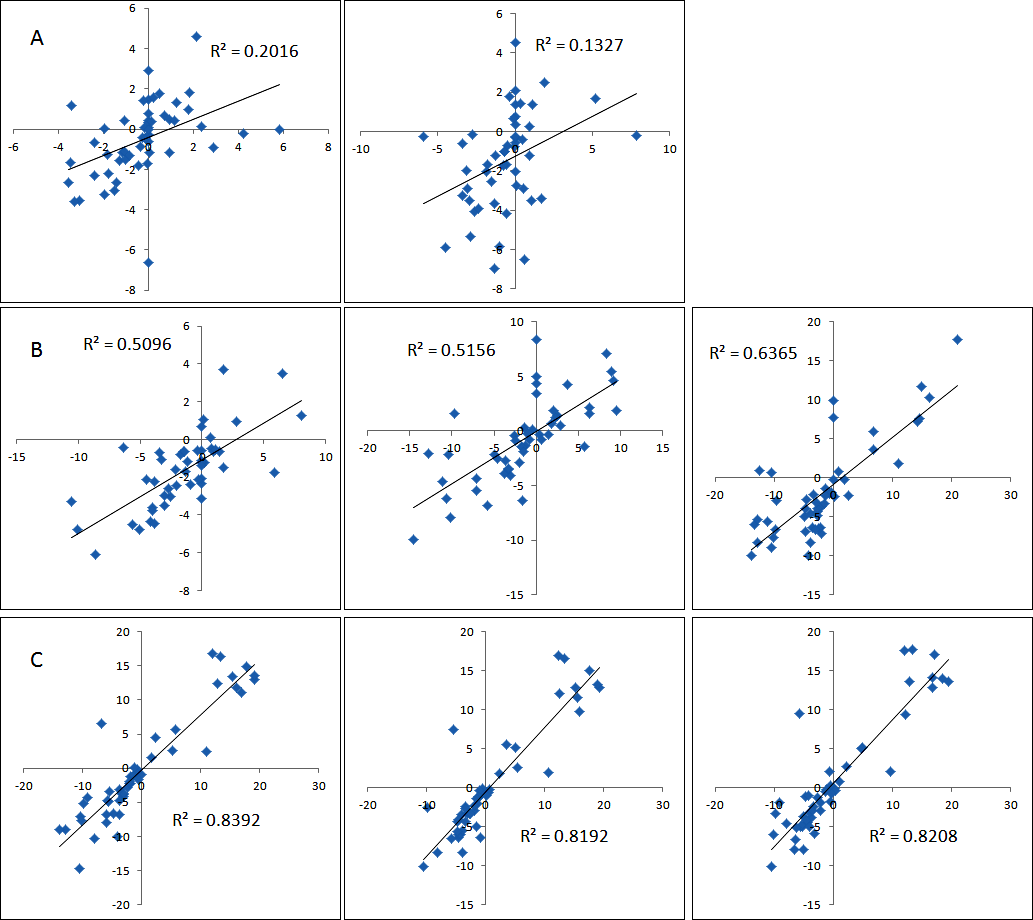

Supplement: Additional file 10 — Figure S2. The correlation of expression levels revealed by RNA-Seq and qRT-PCR. The correlation of RNA-Seq and qRT-PCR during the dedifferentiation process (6 h and 24 h) was relatively low (A), while 48 h NEC and EC time points/stages showed moderate correlation (B). The correlations were higher in the GE, TE and CE stages (C). (TIFF 3140 kb) [file 1471-2229-12-110-S10.tiff]

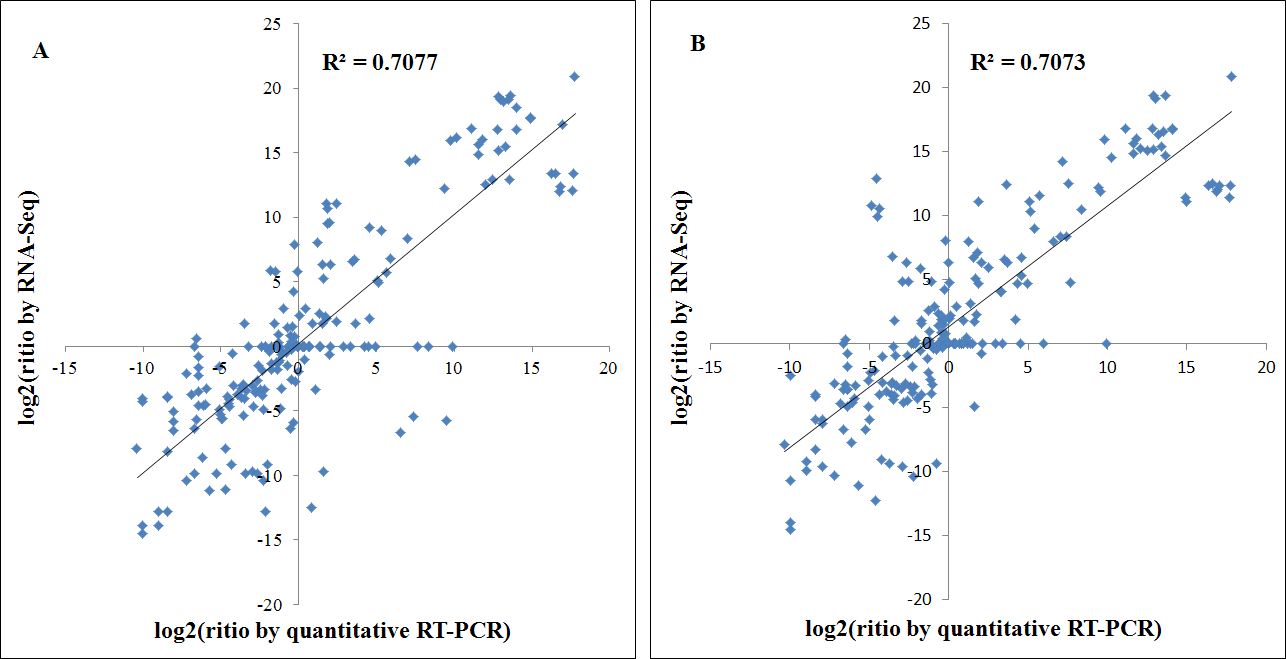

Supplement: Additional file 12 — Figure S3. The correlation expression levels by RNA-Seq and qRT-PCR using two reference databases. The correlation of expression profiles from RNA-Seq and qRT-PCR of 26 randomly selected differentially expressed genes mapped using Reference database 1 (A) and Reference database 2 (B). (TIFF 2790 kb) [file 1471-2229-12-110-S12.tiff]

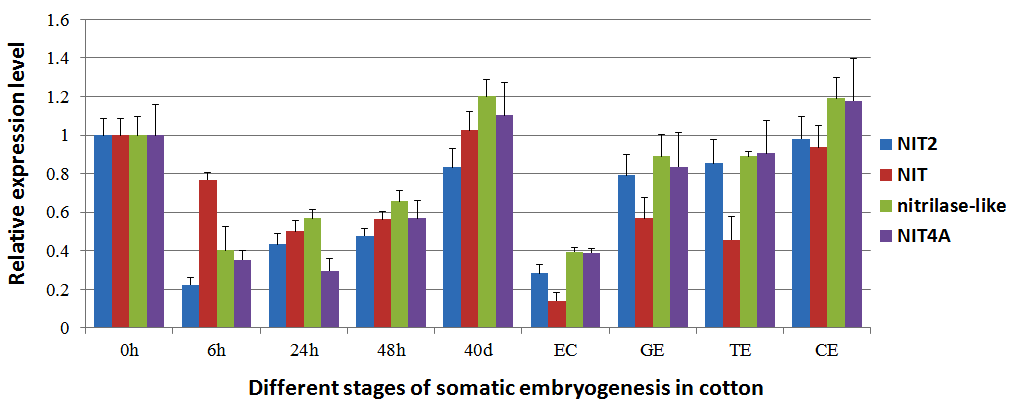

Supplement: Additional file 13 — Figure S4. qRT-PCR analysis of four nitrilases genes derived from cotton database gave the similar expression profile. (TIFF 1361 kb) [file 1471-2229-12-110-S13.tiff]
